# lncRNome: a comprehensive knowledgebase of human long noncoding RNAs

**DOI:** 10.1093/database/bat034

**Published:** 2013-07-11

**Authors:** Deeksha Bhartiya, Koustav Pal, Sourav Ghosh, Shruti Kapoor, Saakshi Jalali, Bharat Panwar, Sakshi Jain, Satish Sati, Shantanu Sengupta, Chetana Sachidanandan, Gajendra Pal Singh Raghava, Sridhar Sivasubbu, Vinod Scaria

**Affiliations:** ^1^GN Ramachandran Knowledge Center for Genome Informatics, CSIR Institute of Genomics and Integrative Biology, Mall Road, Delhi 110007, India, ^2^CSIR Open Source Drug Discovery Unit, Council of Scientific and Industrial Research, Anusandhan Bhavan, Delhi 110001, India, ^3^Genomics and Molecular Medicine, CSIR Institute of Genomics and Integrative Biology, Mall Road, Delhi 110007, India and ^4^Bioinformatics Centre, CSIR Institute of Microbial Technology, Sector 39-A, Chandigarh 160036, India

## Abstract

The advent of high-throughput genome scale technologies has enabled us to unravel a large amount of the previously unknown transcriptionally active regions of the genome. Recent genome-wide studies have provided annotations of a large repertoire of various classes of noncoding transcripts. Long noncoding RNAs (lncRNAs) form a major proportion of these novel annotated noncoding transcripts, and presently known to be involved in a number of functionally distinct biological processes. Over 18 000 transcripts are presently annotated as lncRNA, and encompass previously annotated classes of noncoding transcripts including large intergenic noncoding RNA, antisense RNA and processed pseudogenes. There is a significant gap in the resources providing a stable annotation, cross-referencing and biologically relevant information. lncRNome has been envisioned with the aim of filling this gap by integrating annotations on a wide variety of biologically significant information into a comprehensive knowledgebase. To the best of our knowledge, lncRNome is one of the largest and most comprehensive resources for lncRNAs.

**Database URL:** http://genome.igib.res.in/lncRNome

## Introduction

The availability of technology to annotate transcriptomes at the genome-scale and single-nucleotide resolution has in the recent years provided a new outlook at the transcribed regions within the Human genome (1–3). Contrary to the popular belief, a large number of genomic loci have been presently annotated to be transcriptionally active (4). Many of these regions do not have the potential to encode for functional proteins and thus constitute a class of transcripts, popularly annotated as noncoding RNA (5). The noncoding RNA transcripts have been classified into a number of subclasses, with the most popular classification being based on their size, such as the class of small noncoding RNAs, which include the well-annotated microRNAs (miRNAs) (6), small nucleolar RNAs (snoRNAs), long noncoding RNAs (lncRNAs) and so on.

Long noncoding RNAs (lncRNAs), by definition, are transcripts that are >200 nucleotides in length and do not have the potential to encode for proteins exceeding lengths of ≥30 amino acids (7, 8). Transcriptome annotation in recent years has significantly expanded the repertoire of lncRNAs, not just in humans, but also in other model systems like mouse (9) and zebrafish (10, 11). Although noncoding transcripts with >200 nucleotide lengths have been clubbed together in a general classification of lncRNAs, the members of this class have significant differences in their biological function, genomic loci and regulation. This class includes previously known classes of ncRNAs including the large intergenic noncoding RNA, transcribed pseudogenes, antisense transcripts and several others, including the annotated classes of functionally distinct transcripts such as Xist, which is involved in X inactivation (12) and Hotair (13), involved in epigenetic regulation.

Functionally, the lncRNA class encompasses a wide variety of distinct functions like X-chromosome inactivation, modulation of chromatin structure, regulation of transcriptional and posttranscriptional processes and epigenetic modifications (14). The biological function of lncRNAs is modulated through interaction with other biomolecules in the cell, such as DNA, RNA and proteins (15). Recent evidence has also indicated putative regulatory roles for smaller RNAs processed from lncRNAs, as well as for lncRNAs themselves that harbor regulatory motifs (16, 17). lncRNAs could be regulated in a different way than protein-coding genes (18). Recent evidence also suggests the role of lncRNAs in several diseases including a number of cancers like lung cancers, colorectal and blood neoplasia (7, 19). Candidate lncRNAs like NEAT2 and MALAT1 have been studied in detail with their relations with metastasis in cancers (20–22). Additional candidates like ANRIL have been implicated in diseases like atherosclerosis, (23, 24) while a number of candidate genome-wide association loci map to regions presently annotated as lncRNA genes (25). It has been also suggested that a conceptual understanding of lncRNA as a function of the biological interactions would help to understand disease processes and develop potential drug targets (26).

There are several comprehensive databases for other ncRNAs like miRNAs (27–30), snoRNAs (31); however, there is a paucity of such databases integrating biologically significant annotations for lncRNAs. Although there are lncRNA databases coming up like lncRNAdb (32), NONCODE (33), etc., the extent of lncRNA annotations still remains stringent. lncRNome has been formulated to integrate annotations on a wide variety of biologically significant information into a comprehensive knowledgebase. To the best of our knowledge, lncRNome is one of the largest catalogs for lncRNAs till date, and is available online at the URL: http://genome.igib.res.in/lncRNome.

## Database design and architecture

The lncRNome database has been designed keeping in mind both experimental and computational biologists, so as to provide ready access to biologically relevant data as per the needs of a user. To this end, the structure was designed following consultation with a number of experimental and computational biologists. We created the database to serve as a comprehensive, user-friendly and biologically relevant knowledgebase on human lncRNAs built on MySQL 5.6 and having a PHP-based web interface. In brief, each lncRNA gene has a single page with basic linkouts to other relevant databases, annotation sets and relevant categories of information linked in tabs. Five categories of information are presently available linked with each lncRNA, which includes (i) General Information, (ii) Sequence and Structure, (iii) Interactions and Processing, (iv) Variations and Conservation and (v) Epigenetic Modifications. These categories are connected to the genome browser along with the conservation scores of all lncRNA transcripts (Supplementary File S1).

The category ‘General Information’ hosts information like the gene name, Ensembl gene ID, gene type, gene status, Ensembl transcript ID, transcript name, transcript type, transcript status, chromosome, strand and genomic loci, all of which have been fetched from Gencode release 12 (http://www.gencodegenes.org) (34). The gene names were used to map the HGNC ID, Refseq ID, Havana gene ID, Havana transcript ID, NCBI ID and chromosomal loci from HUGO Gene Nomenclature Committee website (35). The length was calculated using the genomic loci. The details about lncRNA description, disease associations, interactions, overexpression and references were manually curated through literature. The alternate transcripts were derived using in-house scripts and all lncRNAs were provided stable internal IDs.

The lncRNA sequences were downloaded from UCSC Genome Browser Database (36), and the structures were predicted using RNAfold version 1.8.5. Both the parenthesis structure and the minimum free energy structure predicted using the default parameters have been provided.

The third category comprises lncRNA interactions with proteins and other RNAs, lncRNA processing, predicted open reading frames (ORFs) and various motifs. The database hosts 937 quadruplex and 40 hairpins motifs present in lncRNAs. Both the motifs have been predicted using tools developed ‘in-house’, Quadfinder (37) and HairpinFetcher, respectively. It also hosts 3716 miRNA binding sites on lncRNA. More than 10 000 binding sites for nine other proteins, which have been summarized in the section ‘Datasets and Features’, have been provided. These datasets have been mapped using PAR-CLIP (38) and CLIP-Seq datasets as described in the later sections. There are 6808 predicted protein-binding sites also provided in the database, which were predicted using Support Vector Machine–based evaluation of interaction propensities. The 1692 small RNA processing sites have also been provided as described in the sections below. The fourth category consists of 345 351 genomic variations mapped to lncRNAs. The database of single nucleotide polymorphisms (dbSNP) SNPs were downloaded from UCSC genome browser and mapped to lncRNAs. Conservation scores of 66 573 sites within lncRNAs have been provided in this category. The fifth category provides 11 790 epigenetic marks in the promoters of lncRNAs. The datasets were downloaded from the NIH Human Epigenome Roadmap project and mapped to lncRNA promoters. The detailed methods are available as Supplementary methods.

The database also features a comprehensive search option, which enables users to search through lncRNome using different keywords, such as, lncRNA names, Ensembl IDs, known targets, SNPs, diseases, etc. In addition, a separate browse option also allows users to browse the database through either using the chromosome numbers or different lncRNA biotypes. The database also features a genome browser, which can be used to browse through the genome for representative features and also provides a visual representation of the associated genomic annotations available within the database mentioned above along with the conservation scores of lncRNAs.

## Datasets and features

### Long noncoding RNA annotations

**Figure 1.**
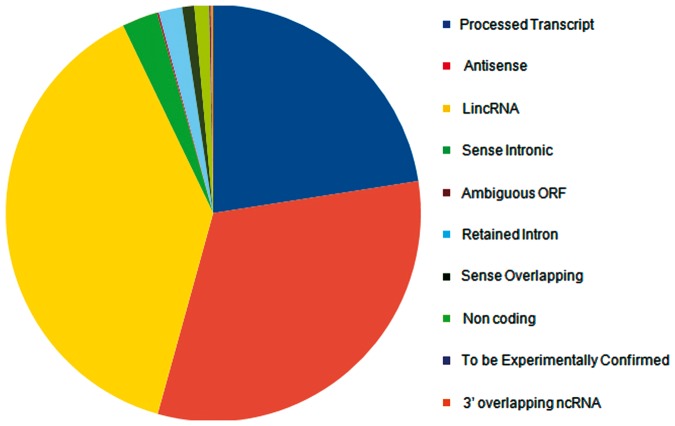
Distribution of Gencode release 12 lncRNAs according to different biotypes.

**Figure 2.**
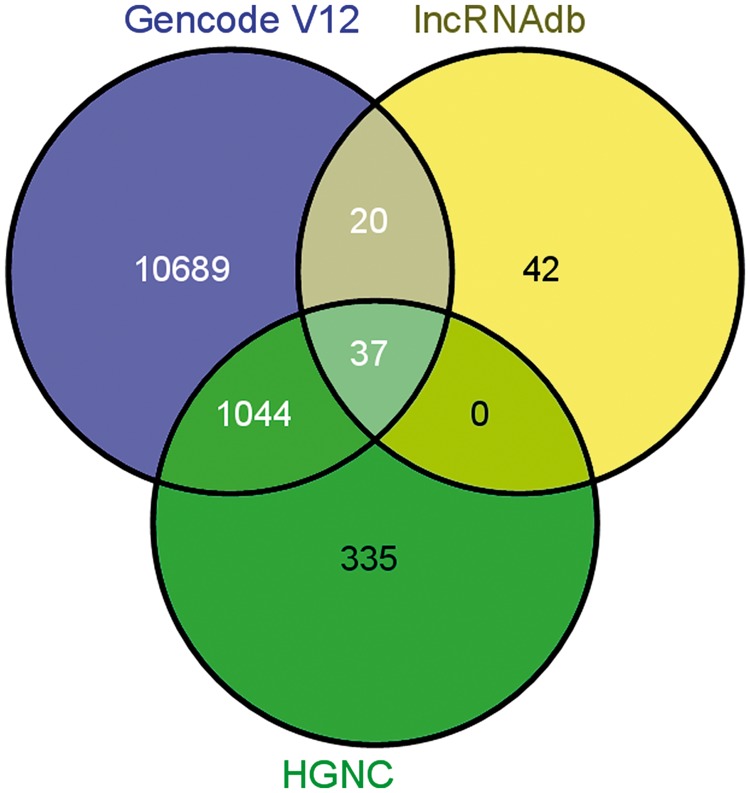
Comparison of annotations between other databases/datasets on long noncoding RNAs.

lncRNA annotations were derived from Gencode release 12 (http://www.gencodegenes.org) (34), which consists of 11 790 lncRNA genes and 18 855 transcripts. The lncRNAs transcripts are classified into 10 different biotypes, the statistics of which has been provided in the Figure 1. In addition, the datasets of lncRNAs and their HGNC IDs were derived from the Human Gene Nomenclature Committee website (35), which consisted of 1073 lncRNAs. Additional mappings were derived for 99 human lncRNAs from lncRNAdb and from literature through manual curation and overlapped with each other based on genomic coordinates (Figure 2). A stable internal ID is also provided for easy access and to enable cross-referencing between the different IDs regularly used by different sequence databases. The consensus IDs forms the primary reference key within lncRNome and has also been used to reference alternate transcript isoforms. Wherever appropriate, all lncRNAs have also been linked back to relevant databases such as Ensembl, HGNC and NCBI for quick cross-reference.

The manual annotation of the functionally characterized lncRNAs is provided, which includes information about the disease associations, expression and functional significance. The annotations are collected from literature surveys and manual curations.

### Sequence and structure and motifs

The lncRNA sequences were downloaded from UCSC Genome Browser using genomic locations of individual transcripts (36). RNA structures were computed using RNAfold with default parameters, which is part of the Vienna RNA package version 1.8.5. Our group has previously suggested the presence of G-quadruplex motifs in lncRNAs that could have potential regulatory functions (39). To enable researchers to further take up experiments in this area, predictions of potential G-quadruplex forming motifs in entire lncRNA transcripts predicted using Quadfinder have been included (37), as well as potential hairpin structures in the lncRNA have been identified using HairpinFetcher.

### lncRNA processing

A recent study conducted by our lab has pointed to a subset of lncRNAs, which could be potentially processed to small RNAs having downstream regulatory functions by having a dual transcriptional output (40). The same analysis was replicated on the present large datasets of lncRNAs. In brief, smallRNA clusters were derived from DeepBase (41), a comprehensive database of smallRNA annotations derived from smallRNA sequencing experiments available in the public domain and overlaid on the lncRNA annotations to derive information on potential lncRNAs that could be processed to smallRNAs.

### Protein-RNA interactions

Recent high-throughput experimental methods for analysis of interactions through pull down and sequencing techniques have provided critical insights into the landscape of protein–RNA interactions in the human genome (42). One of the major datasets of protein–RNA interactions is derived from PAR-CLIP (38) experiments for Argonaute (Ago) proteins, which are critical components of the RISC machinery involved in miRNA targeting. A comprehensive mapping of potential Ago binding sites in the lncRNA transcriptome is provided by mapping the reads to the human transcriptome. Experimental datasets also exist for other proteins including IGF2BP2, IGF2BP3, IGF2P1, PTB, PUM2, QKI, TNRC6A, TNRC6B and TNRC6C, which have also been mapped to the lncRNA transcripts. Because the number of experimental datasets for protein–RNA interactions is scarce, we also incorporated a computational prediction method involving Support Vector Machine–based prediction of residues in RNA, which could have probable propensity to interact with proteins (Panwar and Raghava 2012, unpublished results).

### Genomic variations and conservation

Genome-wide association studies in the recent past have suggested disease associations, which could be modulated by lncRNAs (43). In addition, a number of genomic loci previously shown to be associated with diseases have now been indicated to fall within lncRNA gene loci. To facilitate further in-depth analysis and experimental validation of effect of variations on lncRNA, we have included a comprehensive mapping of genomic variations in lncRNA loci. In brief, the variations corresponding to dbSNP 135 were downloaded (44) and mapped to respective genomic locations of lncRNAs. In addition, disease associated variations were derived from the NIH Catalog of published genome-wide association studies and mapped to respective rsIDs. The PhastCons conservation scores were downloaded from UCSC and the genomic loci were mapped to lncRNAs (45).

### Epigenetic modifications

A recent report from our group suggests that the promoters of lncRNAs could be potentially regulated by mechanisms that are distinct from protein-coding genes supporting the role of lncRNAs in epigenetic regulation of genes. To capture the epigenetic marks, in terms of DNA methylation and histone marks, we have provided a comprehensive access to epigenetic marks in the promoters of lncRNAs. Briefly the raw datasets were downloaded from the NIH Human Epigenome Roadmap project and mapped and analyzed as described in Sati *et al.* (46). The epigenetic marks are also available for browsing through the genome browser. The datasets and genomic mappings are compiled in Table 1.

**Table 1.** Total fields in the database along with the genomic loci mapped

Serial No.	Database fields	Total genomic loci mapped
1	Total lncRNAs	18 855
2	Hairpins	40
3	Methylation and histone modifications	11 790
4	miRNA binding sites	3716
5	Quadruplexes	937
6	Predicted protein-binding sites on lncRNA	6808
7	Small RNA clusters	
8	Single nucleotide polymorphisms	295 851

### Predicted peptides

The open reading frames were predicted for all the lncRNAs using the Sixpack (http://www.ebi.ac.uk/Tools/st/emboss_sixpack/) tool from EMBOSS. The tool translates the given sequence in six frames and peptides starting with Methionine and with length ≥10 amino acids.

## Conclusions and future perspectives

lncRNome is designed to primarily serve as an evidence-based resource of lncRNAs and their functionality in humans. To this end, we have provided stable reference IDs for lncRNA genes and alternate transcript isoforms of a gene with cross-references to other sequence and annotation databases to ensure interoperability and stable referencing. The knowledge base integrates biologically oriented datasets and resources on lncRNA and manual annotations wherever applicable with the aim of providing a one-stop solution for annotation information on lncRNAs.

The interface allows an easy access to various features of lncRNAs comprised within five categories and their sublevels (Supplementary Figure S1). The category ‘General’ provides all the basic annotations of each lncRNA including genomic loci, the associated diseases and various linkouts. The sequences and the predicted structure of the lncRNA are provided in the category ‘Sequence and Structure’. The lncRNA structures are poorly understood and it becomes indispensable to characterize the structures to elucidate the structure–function relationships. Specific lncRNA structures are essential for binding to proteins, RNA and other biomolecules, and to have a better mechanistic insight of lncRNA function, elucidation of its structure becomes important. lncRNome provides information of various hairpin and quadruplex motifs in lncRNAs found to be essential for regulation of a lot of biological processes. Both experimental and prediction datasets on RNA–protein interactions have been provided for lncRNAs revealing various protein and RNA interacting partners of lncRNAs (Supplementary Figure S1). Although the exact mechanism how the lncRNA interacts with different partners is still not known, our data provide a startup point to the community to understand the various regulatory interactions of lncRNAs with their respective partners. Genomic variations in lncRNAs have been studied to understand the effect of SNPs on biogenesis and functions of lncRNAs. The disease-associated SNPs present in lncRNAs might provide information about genotype to phenotype associations. The distribution of epigenetic marks like DNA methylation and histone modifications across transcription start site (TSS) of lncRNAs might help in evaluating the effect of chromatin modifications on gene expression (Supplementary Figure S1).

Because the field is emerging and many more lncRNAs are being discovered and annotated, thanks to the availability of a large number of transcriptome sequencing datasets in public domain, lncRNome in the present form has many gaps. The primary gap being the paucity of information on expression of lncRNAs in different tissues. With the availability of genome-wide transcriptome annotation of many tissues in the public domain, we would enrich the database with this information. We intend to collaborate with other international consortiums to enable cross-linking and sharing of resources seamlessly. In future, we envisage the database to be available as a community-curated and semantically linked interoperable data resource.

## Supplementary Data

Supplementary data are available at *Database* Online.

Supplementary Data
