# Supplementary material for: lncRNome: a comprehensive knowledgebase of human long noncoding RNAs
Source: Database (Oxford). 2013 Jul 11;2013:bat034. doi: 10.1093/database/bat034 (PMC3708617; doi:10.1093/database/bat034)
Supplement: Supplementary Data [file supp_2013_bat034_index.html]

Supplementary Data 

# lncRNome: a comprehensive knowledgebase of human long noncoding RNAs

## 

files

**Files in this Data Supplement:**

- Supplementary Data - jpg file
- Supplementary Data - docx file
