# Supplementary figures and images for: lncRNome: a comprehensive knowledgebase of human long noncoding RNAs
Source: Database (Oxford). 2013 Jul 11;2013:bat034. doi: 10.1093/database/bat034 (PMC3708617; doi:10.1093/database/bat034)

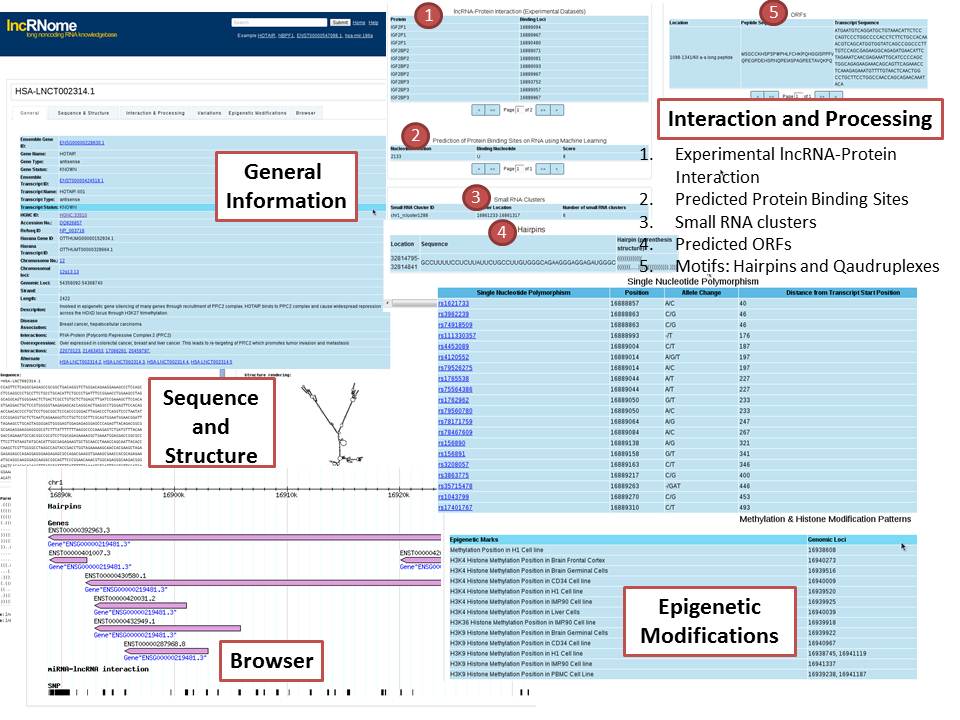

Supplement: Supplementary Data [file supp_bat034_Supp_Fig1.jpg]
