# Supplementary material for: lncRNome: a comprehensive knowledgebase of human long noncoding RNAs
Source: Database (Oxford). 2013 Jul 11;2013:bat034. doi: 10.1093/database/bat034 (PMC3708617; doi:10.1093/database/bat034)
Supplement: Supplementary Data [file supp_bat034_Supplementary_Methods.docx]

**Supplementary Methods File 1.**

**Parameters for Motif Prediction**

*Quadfinder*:

G-stretch: 3 =< x <=5

Loop length: 1<=y<=7

Patterns: GxNy1GxNy2GxNy3Gx, where x denotes the G-stretch and y1, y2 and y3 denote the loop lengths

**Parameters for RNA structure prediction**

*RNAfold*: With default parameters.

Partition function: 1

Maximum expected accuracy: 1

Temperature: 37^o^ C

Circular rna: Off

No tri, tetra or hexaloop hairpins

Dangling end energy: 2

Lonely pairs: off

No GU pairing

No GU pairing at end of helices

**Parameters for analysis of Protein-RNA Interactions**

*Prediction*: Support Vector Machine based prediction model which calculate protein interacting and non-interacting nucleotides.

*Experimental Datasets*: PAR-CLIP read sequences for Ago1-4 of HEK293 cells were mapped to human genome (hg19) using MAQ with stringent criteria allowing no mismatches. The mappings were parsed to identify high-quality miRNA recognition elements using custom scripts having 5 x coverage. These clusters obtained for all the Agos were merged and mapped to lncRNA exons and mRNA exons.

**Parameters for analysis of Small RNA processing**

The small RNA clusters retrieved from DeepBase database were mapped to lncRNA exons from lncRNAdb using bespoke script written in perl. The small RNAs mapping entirely and in same strand orientation to the lncRNA exons were used for further analysis in order to obtain the expression data for the mapped clusters at tissue level.

**Parameters for prediction of Open reading frames**

*Sixpack*:

Table 0: use standard translation code

Firstorf: Do not count the beginning of a sequence as a possible orf, even if it is not finishing with a stop or inferior to the minimal orf size

Lastorf: Do not count the end of a sequence as a possible orf, even if it is not finishing with a stop or inferior to the minimal orf size

Methionine start: no

Do not find on reverse strand

Orfminsize: 1

**Parameters for analysis of Genomic Variations**

Gencode v12 lncRNAs were mapped to validated SNPs from dbSNP 135.

**Parameters for analysis of Epigenetic Modifications**

Aligned files of methylation data and histone modification datasets were downloaded from NIH Roadmap Epigenomic project. Peak was detected using Model-based Analysis for ChIP-Seq (MACS) (version 1.4.0 beta) which was run using default parameters on the files. The summit peaks generated were mapped onto lncRNA loci.
